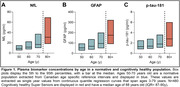# Insights From A Super Seniors Population: Plasma Biomarkers Increase In Magnitude And Variability With Age

**DOI:** 10.1002/alz.086987

**Published:** 2025-01-09

**Authors:** Jennifer G Cooper, Sophie K Stukas, Mohammad Ghodsi, Stephen Leach, Angela Brooks‐Wilson, Cheryl L Wellington

**Affiliations:** ^1^ University of British Columbia, Vancouver, BC Canada; ^2^ Michael Smith Genome Sciences Centre, Vancouver, BC Canada

## Abstract

**Background:**

Plasma biomarkers such as neurofilament light (NfL), glial fibrillary acidic protein (GFAP), and phosphorylated tau‐181 (p‐tau‐181) have been thoroughly investigated in Alzheimer’s disease and other dementias. However, further investigations into how biological variables can influence biomarker concentrations in both normative and cognitively resilient populations will be important for more robust interpretations of biomarker levels. This study investigates how ageing modifies plasma biomarkers in a normative, epidemiologically representative Canadian population and in cognitively healthy seniors over 80 years old.

**Method:**

Biomarkers were analysed on the Quanterix Simoa HD‐X analyzer using Neurology 4‐plex E and p‐tau‐181 assays. N=900 Canadian Health Measures Survey plasma specimens were analyzed as a normative population. Using smoothed quantile regression, the 5th, 50th and 95th percentiles from ages 3‐79 years were determined. The percentiles from 50, 60, and 70 years old were extracted here for analysis. N=480 plasma specimens were analyzed from cognitively healthy Super Seniors Study participants with a median age of 88 years old (IQR= 87‐90y), who had never been diagnosed with dementia, cancer, diabetes, cardiovascular or major pulmonary disease.

**Result:**

In the normative population, between 50 to 70 years old, median concentrations increased by 88% for NfL, 79% for GFAP, and 38% for p‐tau‐181. When compared to 50‐year‐olds in the normative population, the median biomarker concentration in Super Seniors were also higher; 3.4 times for NfL, 3.3 times for GFAP, and 2.2 times for p‐tau‐181. The variability of plasma biomarkers, measured as the range between the 5th and 95th percentiles, also increased with age. Compared to 50 years old, the ranges at age 70 were 2.8 times larger for NfL, 2.2 times larger for GFAP, and 1.5 times larger for p‐tau‐181; While the ranges observed in Super Seniors, compared to 50 years old, were 5.3 times larger for NfL, 3.4 times larger for GFAP, and 2.8 times larger for p‐tau‐181.

**Conclusion:**

Cognitively healthy seniors over 80 years have higher and more variable plasma biomarker concentrations than a normative 50‐year‐olds. These data indicate that age, regardless of cognitive status, modifies biomarker concentrations over the age of 80 years.